# Supplementary material for: Multiple levels of transcriptional regulation control glycolate metabolism in Paracoccus denitrificans
Source: mBio. 2024 Jul 2;15(8):e01524-24. doi: 10.1128/mbio.01524-24 (PMC11323563; doi:10.1128/mbio.01524-24)
Supplement: Supplemental Information — Supplemental figures and tables. [file mbio.01524-24-s0001.pdf]

Supplementary Information for

Multiple levels of transcriptional regulation control glycolate metabolism in *Paracoccus denitrificans*

Lennart Schada von Borzyskowski[1,2] *, Lucas Hermann[3], Katharina Kremer[1], Sebastian Barthel[1], Bianca Pommerenke[1], Timo Glatter[4], Nicole Paczia[5], Erhard Bremer[3,6], Tobias J. Erb[1,6] *

[1]Department of Biochemistry & Synthetic Metabolism, Max Planck Institute for Terrestrial Microbiology, Karl-von-Frisch-Str. 10, D-35043 Marburg, Germany; [2]Institute of Biology Leiden, Leiden University, Sylviusweg 72, 2333 BE Leiden, The Netherlands; [3]Laboratory for Microbiology, Department of Biology, Philipps-University Marburg, Karl-von-Frisch-Str. 8, D-35043 Marburg, Germany; [4]Facility for Mass Spectrometry and Proteomics, Max Planck Institute for Terrestrial Microbiology, Karl-von-Frisch-Str. 10, D-35043 Marburg, Germany; [5]Facility for Metabolomics and Small Molecule Mass Spectrometry, Max Planck Institute for Terrestrial Microbiology, Karl-von-Frisch-Str. 10, 35043 Marburg, Germany; [6]LOEWE-Center for Synthetic Microbiology, Philipps-University Marburg, Karl-von-Frisch-Str. 8, D-35043 Marburg, Germany.

** corresponding authors: L.S.v.B. (L.Schada.von.Borzyskowski@biology.leidenuniv.nl), T.J.E. (toerb@mpi-marburg.mpg.de)*

Table of Contents Supplementary Information:

Supplementary Figure 1 .... *Page 2*
Supplementary Figure 2 .... *Page 3*
Supplementary Figure 3 .... *Page 4*
Supplementary Figure 4 .... *Page 5*
Supplementary Figure 5 .... *Page 5*
Supplementary Figure 6 .... *Page 6*
Supplementary Figure 7 .... *Page 7*
Supplementary Figure 8 .... *Page 8*
Supplementary Figure 9 .... *Page 9*
Supplementary Table 1 .... *Page 10*
Supplementary Table 2 .... *Page 11*
Supplementary Table 3 .... *Page 12*
Supplementary Table 4 .... *Page 12*
Supplementary Table 5 .... *Page 13*
Supplementary Table 6 .... *Page 14*
References .... *Page 15*

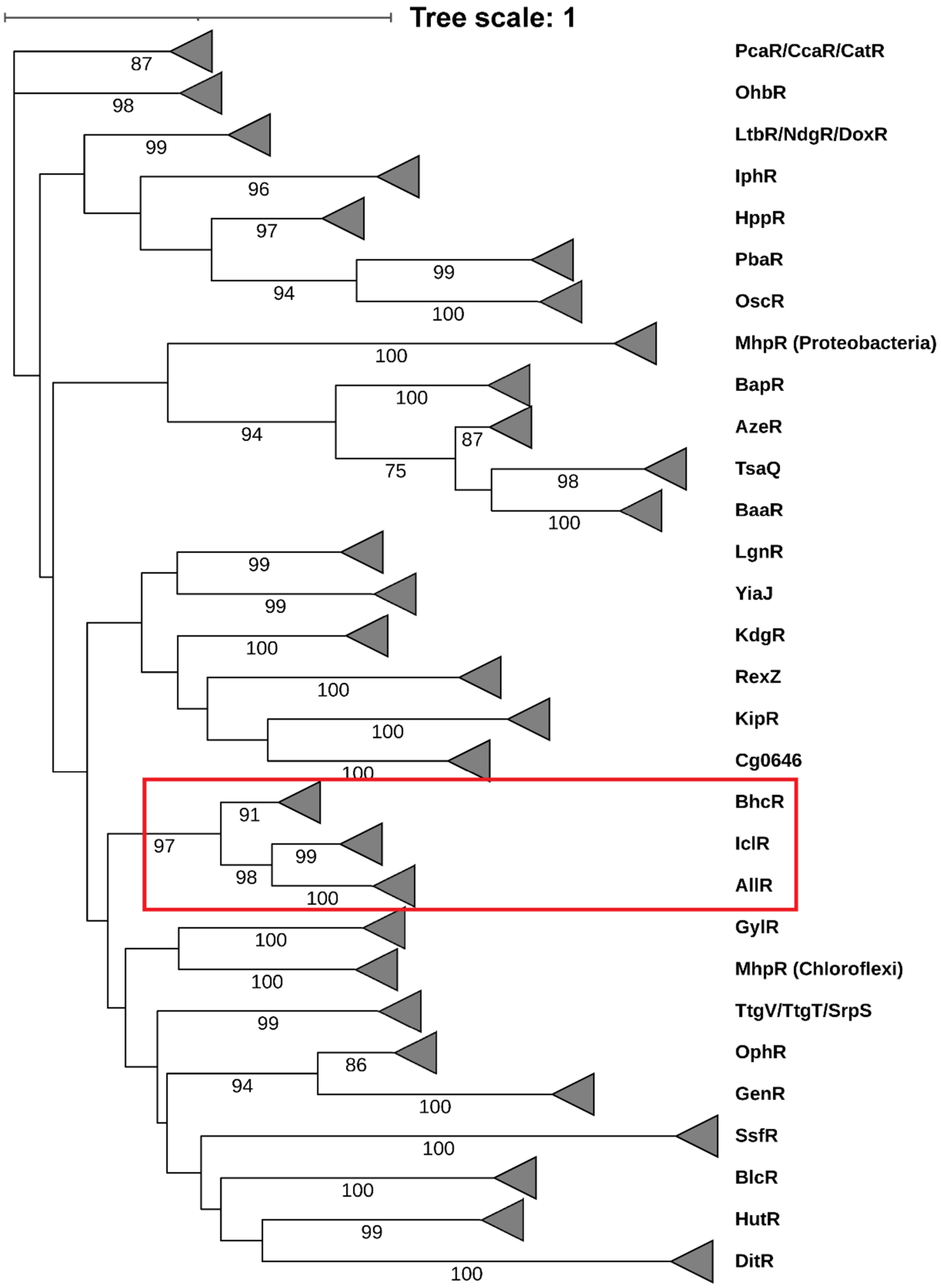

Supplementary Figure 1: Maximum likelihood phylogenetic tree of the IclR family of transcriptional regulators. Sequences of the transcription factor BhcR and its homologs form a distinct clade within the branch of glyoxylate-binding transcription factors (denoted by a red frame), which also includes the AllR and IclR subfamilies. Bootstrap values of at least 50 are given on the respective nodes.

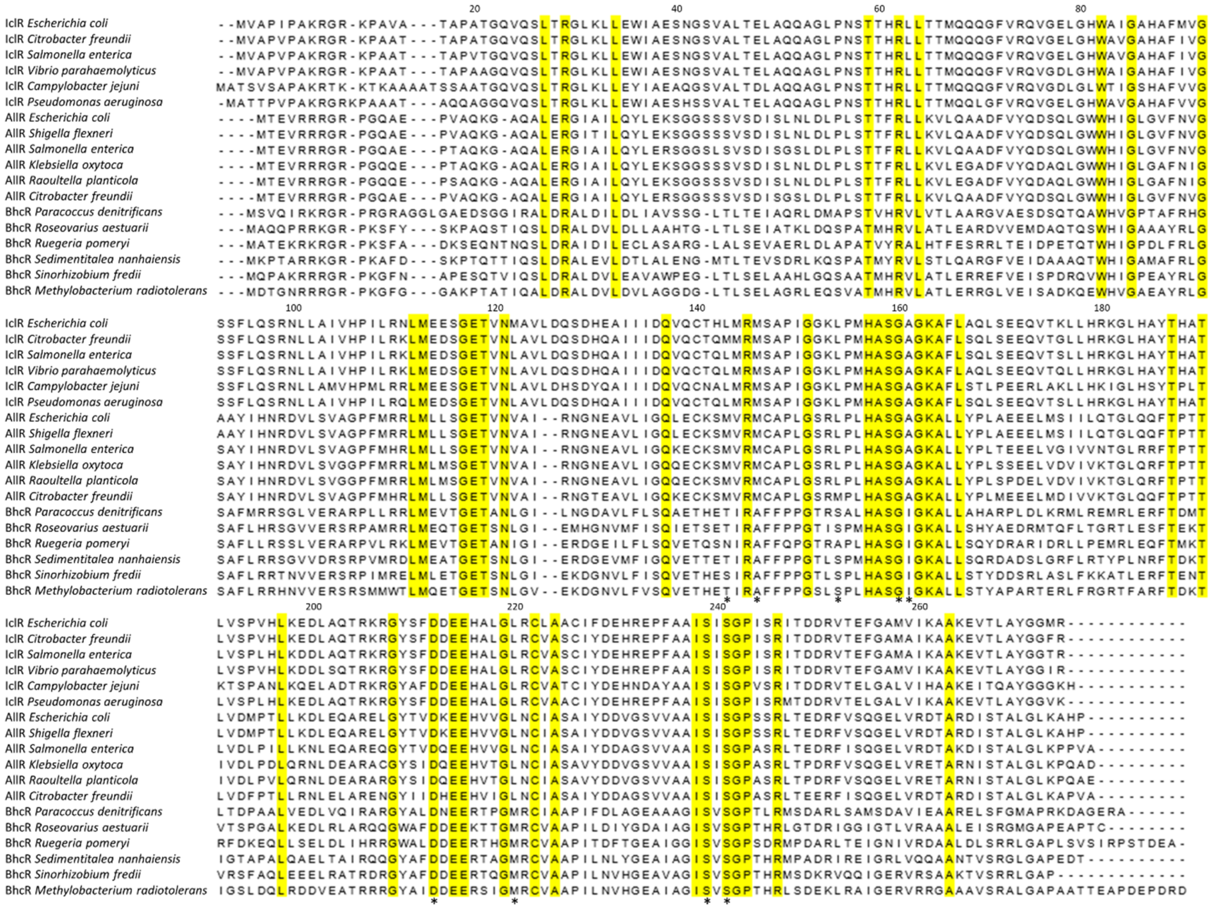

Supplementary Figure 2: Alignment of IclR, AllR, and BhcR amino acid sequences. Amino acids that are fully conserved across this dataset are highlighted in yellow. Amino acids that were previously identified as ligand-binding residues in IclR are denoted with asterisks below the alignment. Numbering of amino acids above the alignment is based on the sequence of *E. coli* IclR.

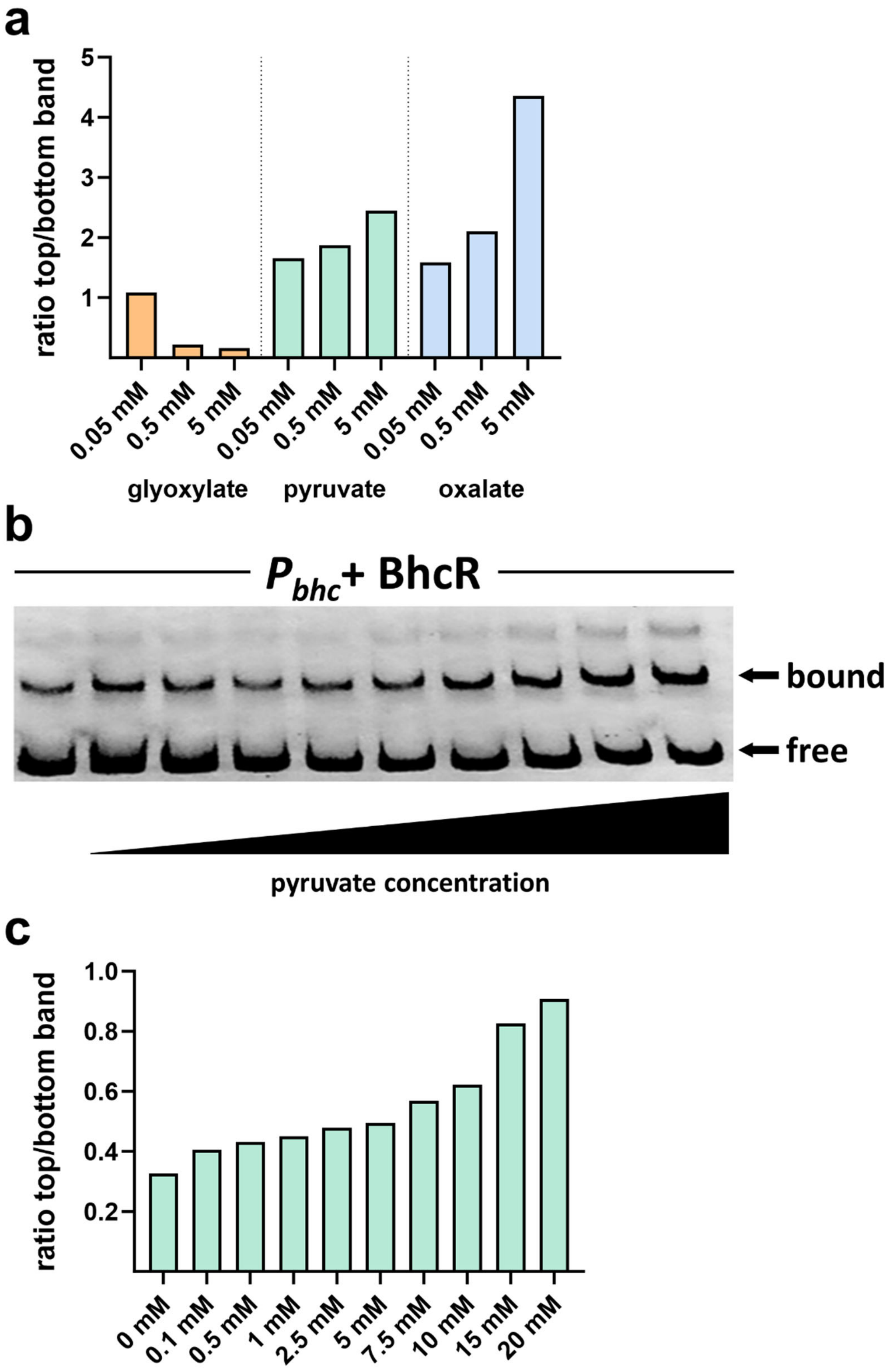

Supplementary Figure 3: DNA-binding properties of BhcR. a, Quantification of band intensity ratios of the EMSA shown in **Figure 1a** (bottom). **b**, The P_{bhc}–BhcR complex (10,000x molar excess BhcR) was incubated with increasing concentrations (0.1 mM; 0.5 mM; 1 mM; 2.5 mM; 5 mM; 7.5 mM; 10 mM; 15 mM; 20 mM) of pyruvate and subsequently separated by electrophoresis to assess the effect of this metabolite on complex formation. Increasing concentrations of pyruvate increase the binding of BhcR to the P_{bhc} DNA fragment. **c**, Quantification of band intensity ratios of the EMSA shown in **b**.

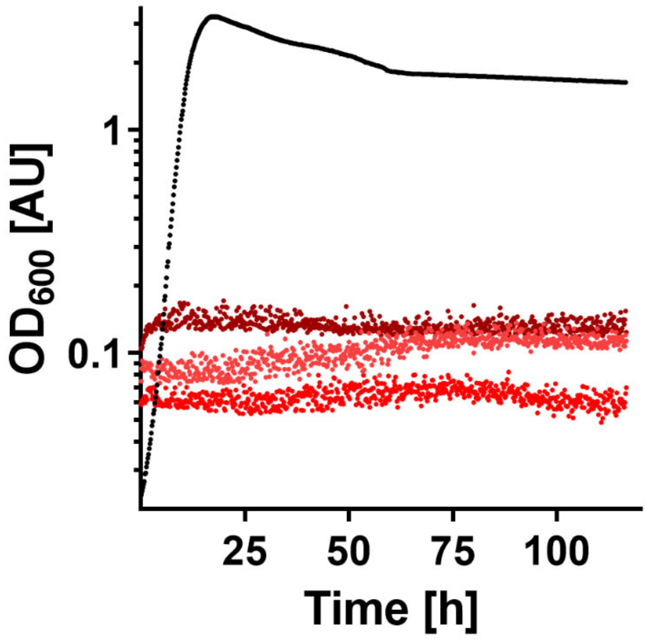

Supplementary Figure 4: *Paracoccus denitrificans* is not capable of growth on oxalate. No growth was observed on 5 mM (light red), 10 mM (red), or 30 mM oxalate (dark red) as sole source of carbon and energy. Growth on 40 mM pyruvate (black) was monitored as a positive control.

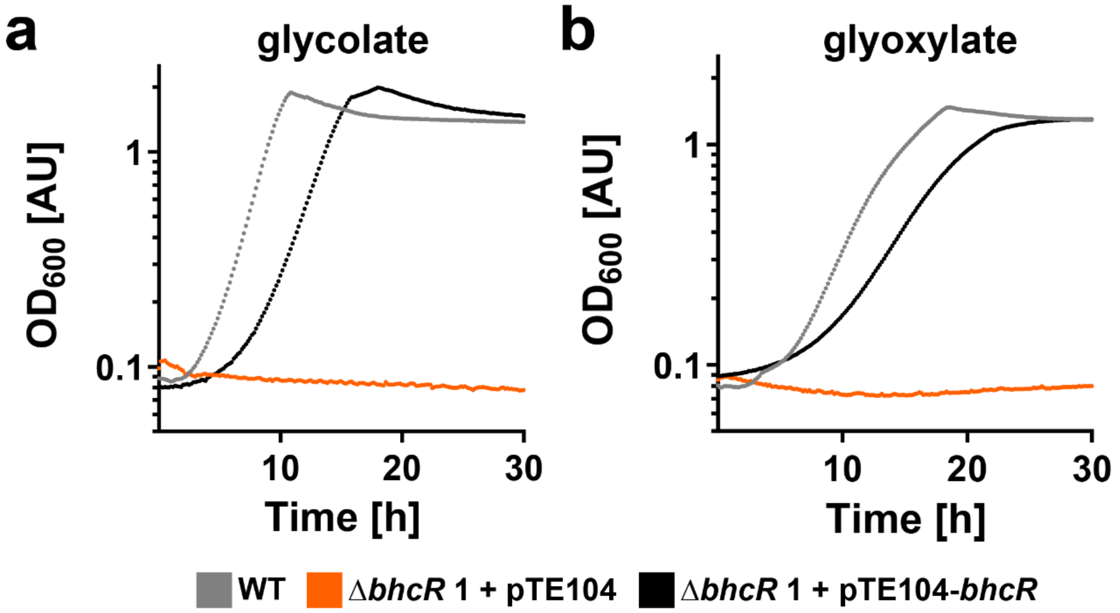

Supplementary Figure 5: Complementation of *P. denitrificans* Δ*bhcR*. **a**, **b**, Growth curves of wild-type *P. denitrificans* DSM 413 (grey), a *bhcR* deletion strain containing a negative control plasmid (orange), and a *bhcR* deletion strain containing a *bhcR* expression plasmid (black) grown in the presence of 60 mM glycolate (**a**) or 60 mM glyoxylate (**b**). Plasmid-based expression of *bhcR* recovers the ability of the *bhcR* deletion strain to grow on glycolate or glyoxylate. These experiments were repeated three times independently with similar results.

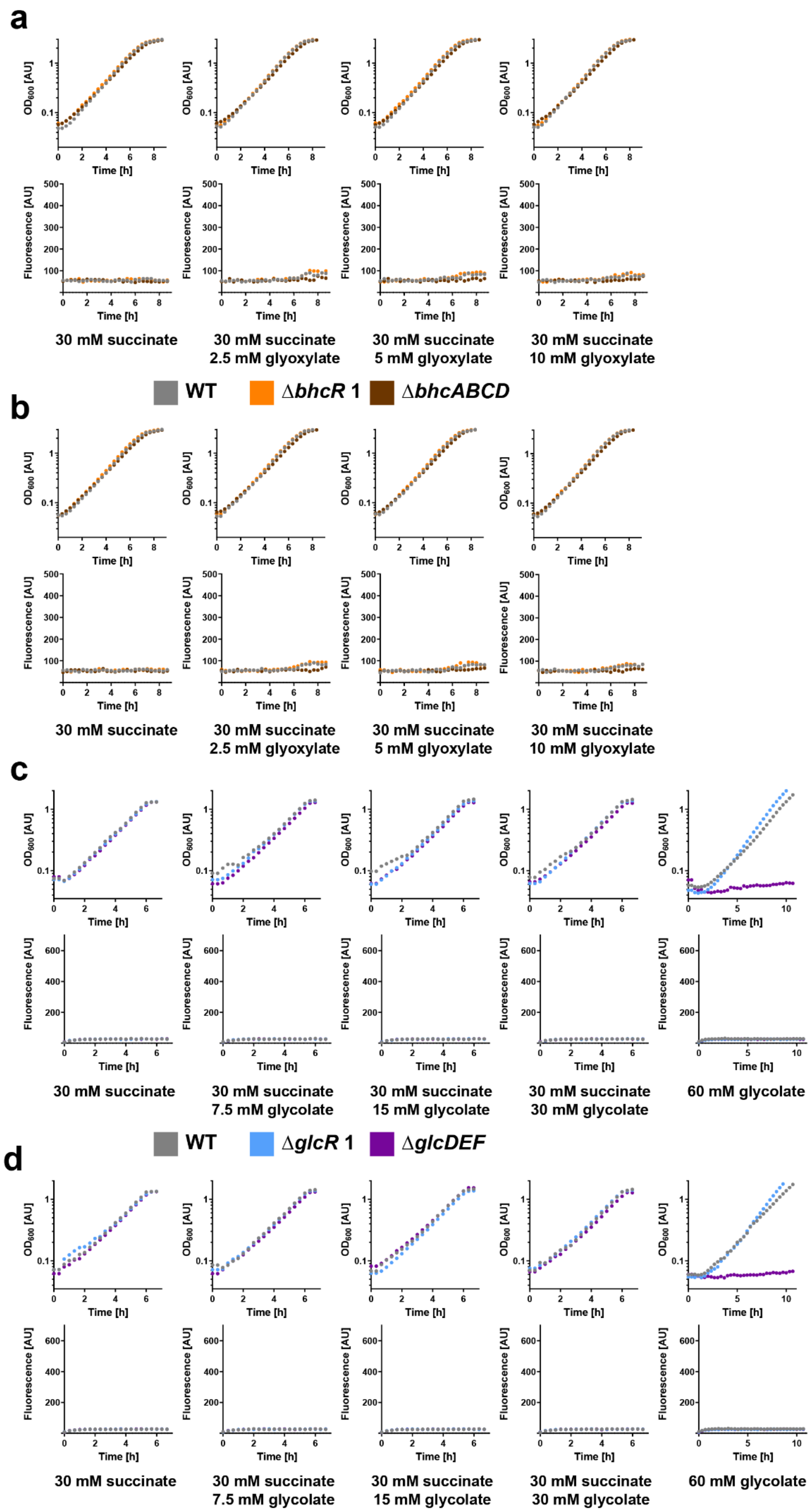
a
b
c
d
OD600 [AU]
Fluorescence [AU]
Time [h]
30 mM succinate
30 mM succinate
2.5 mM glyoxylate
30 mM succinate
5 mM glyoxylate
30 mM succinate
10 mM glyoxylate
WT
ΔbhcR 1
ΔbhcABCD
30 mM succinate
30 mM succinate
7.5 mM glycolate
30 mM succinate
15 mM glycolate
30 mM succinate
30 mM glycolate
60 mM glycolate
WT
ΔglcR 1
ΔglcDEF

Supplementary Figure 6: Characterization of *P. denitrificans* promoter reporter strains (negative controls). a, b, Growth and fluorescence of promoter reporter strains Δ*bhcR* (orange), Δ*bhcABCD* (brown), and WT (grey) without a plasmid (**a**) or with pTE714 (**b**) on different carbon sources. **c, d**, Growth and fluorescence of promoter reporter strains Δ*glcR* (light blue), Δ*glcDEF* (purple), and WT (grey) without a plasmid (**c**) or with pTE714 (**d**) on different carbon sources. All experiments were repeated three times independently with similar results.

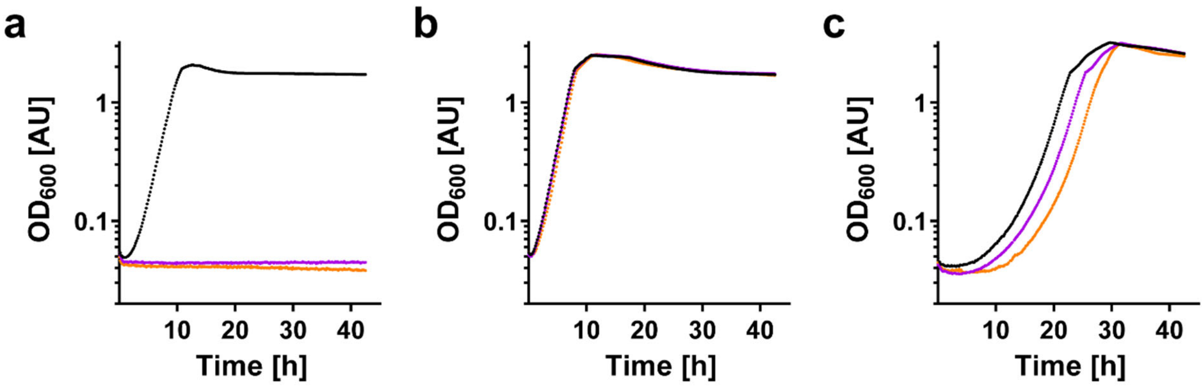

Supplementary Figure 7: Growth of *P. denitrificans* Δ*glcDEF*. Two Δ*glcDEF* deletion strains, in which the genes Pden_4397-99 were replaced with a kanamycin resistance cassette in either the same or the opposite direction of transcription (pink, orange) were unable to grow on 60 mM glycolate (**a**). In contrast, these two deletion strains grew similarly to the WT strain (black) on 30 mM succinate (**b**) and 60 mM acetate (**c**).

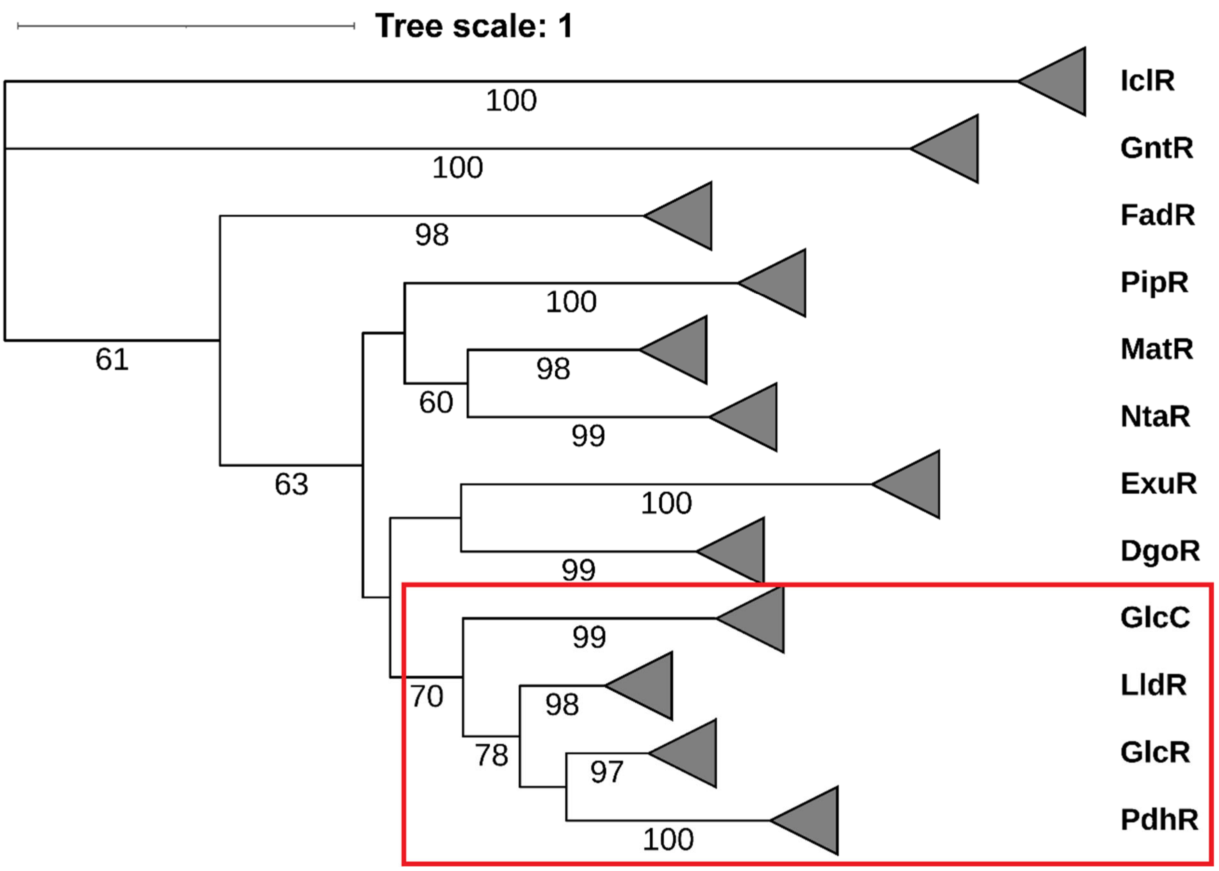

Supplementary Figure 8: Maximum likelihood phylogenetic tree of the FadR subfamily of transcriptional regulators. Sequences of IclR-type and GntR-type regulators were used as outgroups. Sequences of the regulator GlcR and its homologs form a distinct clade within the branch of transcription factors that interact with glycolate, pyruvate, or lactate (denoted by a red frame), which also includes the GlcC, LldR, and PdhR subfamilies. Bootstrap values of at least 50 are given on the respective nodes.

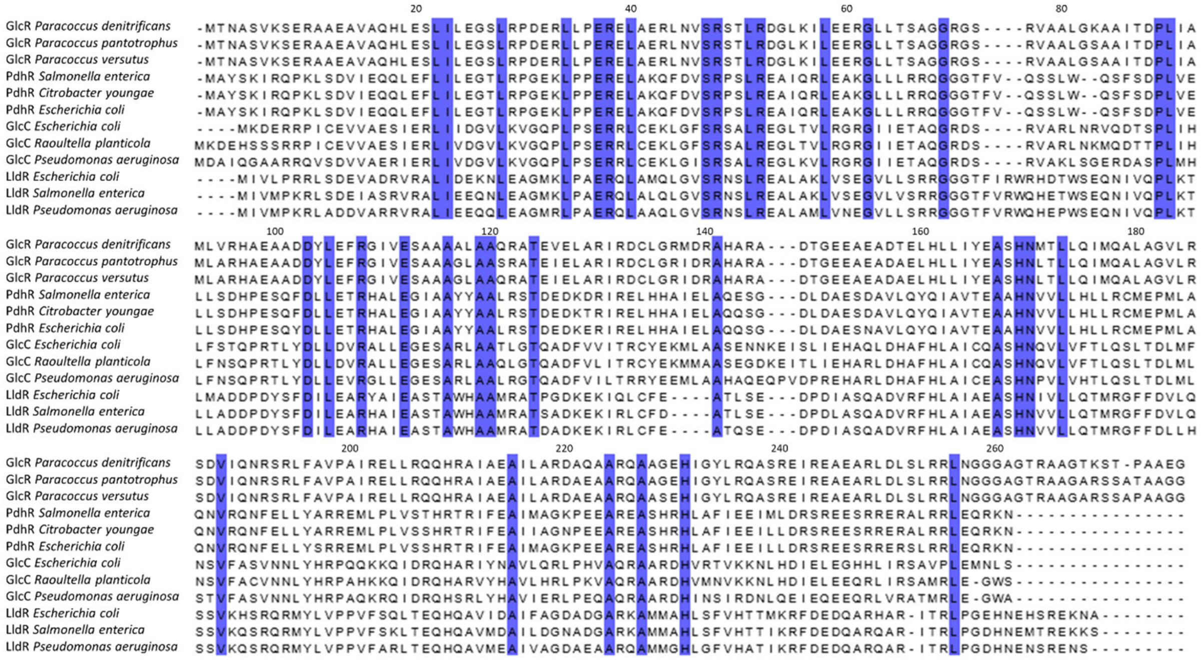

Supplementary Figure 9: Alignment of GlcR, PdhR, GlcC, and LldR amino acid sequences. Amino acids that are fully conserved across this dataset are highlighted in blue. Numbering of amino acids above the alignment is based on the sequence of *P. denitrificans* GlcR.

Supplementary Table 1: The *glcRDEF* gene cluster in various alphaproteobacterial strains. Gene IDs for the respective *glcRDEF* genes are according to the respective genome sequences deposited in the NCBI Nucleotide database (https://www.ncbi.nlm.nih.gov/nucleotide/).

Strain	Order	*glcR*	*glcD*	*glcE*	*glcF*
Paracoccus denitrificans DSM 413	*Rhodobacterales*	Pden_4400	Pden_4399	Pden_4398	Pden_4397
Paracoccus pantotrophus DSM 2944	*Rhodobacterales*	ESD82_RS02595	ESD82_RS02600	ESD82_RS02605	ESD82_RS02610
Paracoccus zeaxanthinifaciens ATCC 21588	*Rhodobacterales*	F804_RS0111220	F804_RS0111210	F804_RS0111205	F804_RS0111200
Paracoccus methylovorus H4-D09	*Rhodobacterales*	JWJ88_RS16105	JWJ88_RS16110	JWJ88_RS16115	JWJ88_RS16120
Methylarcula marina VKM B-2159	*Rhodobacterales*	A7A09_RS08100	A7A09_RS08105	A7A09_RS08110	A7A09_RS08115
Puniceibacterium antarcticum SM1211	*Rhodobacterales*	P775_RS11050	P775_RS11045	P775_RS11040	P775_RS11035
Rhodobacter xinxiangensis TJ48	*Rhodobacterales*	E2K76_RS04305	E2K76_RS04315	E2K76_RS04320	E2K76_RS04325
Chenggangzhangella methanolivorans CHL1	*Rhizobiales*	K6K41_RS14170	K6K41_RS14165	K6K41_RS14160	K6K41_RS14155
Afipia sp. 1NLS2	*Rhizobiales*	AFIDRAFT_RS03560	AFIDRAFT_RS03555	AFIDRAFT_RS03550	AFIDRAFT_RS03545

Supplementary Table 2: The CceR regulon in *P. denitrificans* and *Rhodobacter sphaeroides*. Data for *R. sphaeroides* and the predicted CceR regulon of *P. denitrificans* were previously published (1).

protein (gene)	*R. sphaeroides*	*P. denitrificans* (predicted)	*P. denitrificans* (experimentally determined)
PEP carboxykinase (*pckA*)	X		X
malate dehydrogenase (*mdh*)	X		
succinate dehydrogenase (*sdhDA*)	X		
ATP synthase (*atpBEFHAC*)	X		
fructose 1,6-bisphosphatase (*fbp*)	X		
succinyl-CoA synthetase (*sucCD*)	X		
2-OG dehydrogenase (*sucB*)	X		
fumarase (*fumC*)	X		
NADH dehydrogenase (*nuoL*)	X		
fructose 1,6-bisphosphate aldolase (*fba*)	X		X
pyruvate dehydrogenase (*pdhAB*)	X		
glucose 6-phosphate dehydrogenase (*zwf*)	X		X
6-phosphogluconolactonase (*pgl*)	X		X
glucose 6-phosphate isomerase (*pgi*)	X		X
phosphogluconate dehydratase (*edd*)	X	X	X
KDPG aldolase (*eda*)	X	X	X
cytochrome c-554 (*cycF*)	X		
pyruvate kinase (*pyk*)		X	X
TRAP dicarboxylate transporter (*dctP*)		X	X
gluconate transporter (*glnT*)		X	X
gluconokinase (*glnK*)		X	X
alpha-1,4-glucan phosphorylase (*glgP*)		X	X
1,4-alpha-glucan branching enzyme (*glgB*)		X	X
glucose 1-phosphate adenylyltransferase (*glgC*)		X	X
glycogen synthase (*glgA*)		X	X
glycogen debranching enzyme (*glgX*)		X	X
4-alpha-glucanotransferase (*malQ*)		X	X
phosphogluco/mannomutase (*pgm*)		X	X
phosphofructokinase (*pfk*)			X
glucokinase (*glk*)			X
6-phosphogluconate dehydrogenase (*gnd*)			X
propionyl-CoA synthetase (*prpE*)			X
2-methylisocitrate lyase (*prpB*)			X
2-methylisocitrate synthase (*prpC*)			X
2-methylisocitrate dehydratase (*prpD*)			X
malic enzyme (*maeB*)			X
inositol degradation pathway (Pden_1672-1684)			X

Supplementary Table 3: Calculated and experimentally determined growth rates of *P. denitrificans* on minimal medium with glycolytic and gluconeogenic carbon substrates. The growth rate composition formula used here was previously developed and validated for *E. coli* (2). All experimentally determined growth rates are averages from six independent experiments; variability between independent experiments was not more than 5%. For the calculations, a λ_C value of 0.85 was estimated for *P. denitrificans*.

**Growth rate μ (h^{-1})**	**as sole carbon source**	**with 10 mM glucose (calculated)**	**with 10 mM glucose (experimentally determined)**
**30 mM glycolate**	0.51	0.60	0.51
**30 mM glyoxylate**	0.28	0.48	0.39
**10 mM glucose**	0.38	-	-

Supplementary Table 4 | Strains used in this study

**strain**	**genotype or relevant features**[a]	**source or reference**
E. coli DH5α	*supE44*, *ΔlacU169* (*Φ80lacZDM15*), *hsdR17*, *recA1*, *endA1*, *gyrA96*, *thi*-1, *relA1*	Thermo Fisher Scientific, Waltham, USA
E. coli ST18	*pro*, *thi*, *hsdR1*,Tp^R, Sm^R; chromosome::RP4-2, Tc::Mu-Kan::Tn7/λpir, λpir, Δ*hemA*	Thoma and Schobert (3)
E. coli BL21 AI	*ompT*, *gal*, *dcm*, *lon*, *hsdS*$_B$($r_B^- m_B^-$), [*malB*$^+$]$_{K\text{-}12}$(λ^S), *araB*::*T7RNAP-tetA*	Thermo Fisher Scientific, Waltham, USA
P. denitrificans DSM 413	WT strain	Beijerinck and Minkman (4)
P. denitrificans DSM 413 Δ*bhcR* 1	Δ3922; Km^R (orientation 1)	this work
P. denitrificans DSM 413 Δ*bhcR* 2	Δ3922; Km^R (orientation 2)	this work
P. denitrificans DSM 413 Δ*bhcABCD* 1	Δ3921-18; Km^R (orientation 1)	Schada von Borzyskowski, Severi (5)
P. denitrificans DSM 413 Δ*bhcABCD* 2	Δ3921-18; Km^R (orientation 2)	Schada von Borzyskowski, Severi (5)
P. denitrificans DSM 413 Δ*glcR* 1	Δ4400; Km^R (orientation 1)	this work
P. denitrificans DSM 413 Δ*glcR* 2	Δ4400; Km^R (orientation 2)	this work
P. denitrificans DSM 413 Δ*glcDEF* 1	Δ4399-97; Km^R (orientation 1)	this work
P. denitrificans DSM 413 Δ*glcDEF* 2	Δ4399-97; Km^R (orientation 2)	this work
P. denitrificans DSM 413 Δ*cceR* 1	Δ1978; Km^R (orientation 1)	this work
P. denitrificans DSM 413 Δ*cceR* 2	Δ1978; Km^R (orientation 2)	this work

[a] Km^R, kanamycin resistance; Tp^R, trimethoprim resistance; Sm^R, streptomycin resistance

Supplementary Table 5 | Plasmids used in this study

plasmid	relevant features[a]	source or reference
pET16b	*E. coli* expression vector, T7 promoter, Amp^R	Merck Chemicals GmbH, Darmstadt, Germany
pET16b-BhcR	expression vector for N-terminally His-tagged BhcR, Amp^R	Schada von Borzyskowski, Severi (5)
pET16b-GlcR	expression vector for N-terminally His-tagged GlcR, Amp^R	this work
pMBP-sfgfp_dropout (pTE5400)	expression vector for N-terminally His-tagged-maltose-binding protein (10x-His-MBP) with sfgfp dropout, compatible with Marburg Collection (6), Cam^R	this work
pMBP-GlcR (pTE5418)	expression vector for N-terminally His- and MBP-tagged GlcR, Cam^R	this work
pREDSIX	mobilizable, high-copy-number cloning and mutagenesis vector; Amp^R	Ledermann, Strebel (7)
pRGD-KmR	donor vector for resistance gene, *aphII* in polylinker; Amp^R, Km^R	Ledermann, Strebel (7)
pREDSIX-*bhcR-1*	knockout vector for the *bhcR* gene in *P. denitrificans* DSM 413; Km^R (orientation 1)	this work
pREDSIX-*bhcR-2*	knockout vector for the *bhcR* gene in *P. denitrificans* DSM 413; Km^R (orientation 2)	this work
pREDSIX-gl*cR-1*	knockout vector for the *glcR* gene in *P. denitrificans* DSM 413; Km^R (orientation 1)	this work
pREDSIX-gl*cR-2*	knockout vector for the *glcR* gene in *P. denitrificans* DSM 413; Km^R (orientation 2)	this work
pREDSIX-*cceR-1*	knockout vector for the *cceR* gene in *P. denitrificans* DSM 413; Km^R (orientation 1)	this work
pREDSIX-*cceR-2*	knockout vector for the *cceR* gene in *P. denitrificans* DSM 413; Km^R (orientation 2)	this work
pREDSIX-*glcDEF-1*	knockout vector for the *glcDEF* gene cluster in *P. denitrificans* DSM 413; Km^R (orientation 1)	this work
pREDSIX-*glcDEF-2*	knockout vector for the *glcDEF* gene cluster in *P. denitrificans* DSM 413; Km^R (orientation 2)	this work
pTE100	empty expression vector for Alphaproteobacteria; Tc^R	Schada von Borzyskowski, Remus-Emsermann (8)
pTE714	promoter probe vector for Alphaproteobacteria based on pTE100 containing a RBS and mCherry; Tc^R	this work
pTE714_3922/3921_ig	promoter probe vector containing mCherry under control of the promoter located in the intergenic region between Pden_3922 and Pden_3921; Tc^R	this work
pTE714_4400/4399_ig	promoter probe vector containing mCherry under control of the promoter located in the intergenic region between Pden_4400 and Pden_4399; Tc^R	this work
pTE104	expression vector for Alphaproteobacteria containing the P_{coxB} promoter; Tc^R	Schada von Borzyskowski, Remus-Emsermann (8)
pTE104-*bhcR*	expression vector for Alphaproteobacteria containing the *bhcR* gene under control of the P_{coxB} promoter; Tc^R	this work

[a] Km^R, kanamycin resistance; Amp^R, ampicillin resistance; Tc^R, tetracycline resistance; Cam^R, chloramphenicol resistance

Supplementary Table 6 | Primers used in this study

target	name	sequence[a]	cut site
Pden_*glcR*	glcR_16b_fw	5'-GACGCTG**CATATG**ACCAACGCATCCGTGAAATCC-3'	*Nde*I
Pden_*glcR*	glcR_16b_rv	5'-GACACTC**GGATCC**CTAGCCCTCTGCGGCCGGGGTGG-3'	*Bam*HI
Pden_*bhcR*_up	bhcR_up_fw	5'-GGTCTGACAGGTTTAAACTCTAGACCGGAAATCCATCGAACCGATG-3'	---
Pden_*bhcR*_up	bhcR_up_rv	5'-AAGTTTAGACGAA**GGTACC**TCAATTTTTCCTTTCGACAAC-3'	*Kpn*I
Pden_*bhcR*_down	bhcR_down_fw	5'-AAATTGA**GGTACC**TTCGTCTAAACTTGACCAGGACATGCCC-3'	*Kpn*I
Pden_*bhcR*_down	bhcR_down_rv	5'-CTTAAGGCTAGCATGCATCCTAGGCGGGCTGTAGCCGGGCCCGATC-3'	---
Pden_*glcR*_up	glcR_up_fw	5'-GGTCTGACAGGTTTAAACTCTAGACAGGCCCCATCTCGACCCCAC-3'	---
Pden_*glcR*_up	glcR_up_rv	5'-GGCCAGTCCTCT**CATATG**GGGCGCTCATGCGGTTGTCG-3'	*Nde*I
Pden_*glcR*_down	glcR_down_fw	5'-AGCGCCC**CATATG**AGAGGACTGGCCGGCAAGGAAAAG-3'	*Nde*I
Pden_*glcR*_down	glcR_down_rv	5'-CTTAAGGCTAGCATGCATCCTAGGCGATGGCGACCGTCGCCACCC-3'	---
Pden_*cceR*_up	cceR_up_fw	5'-GGTCTGACAGGTTTAAACTCTAGACTCCGCAAGGCCGGCCAGGAC-3'	---
Pden_*cceR*_up	cceR_up_rv	5'-GCAGGCGCGGCG**CATATG**CGCAACTACCGGGACGC-3'	*Nde*I
Pden_*cceR*_down	cceR_down_fw	5'-AGTTGCG**CATATG**CGCCGCGCCTGCGGGCGGCG-3'	*Nde*I
Pden_*cceR*_down	cceR_down_rv	5'-CTTAAGGCTAGCATGCATCCTAGGCCATGCCAGCTGCATCAGCGTGA ACCATGTCGTCTG-3'	---
Pden_*glcDEF*_up	glcDEF_up_fw	5'-GGTCTGACAGGTTTAAACTCTAGACCCGCATGACCAACGCCACCGTG-3'	---
Pden_*glcDEF*_up	glcDEF_up_rv	5'-AAGGGAGGAGAGC**CATATG**GCTCGCCCCGGCTTGGGAACG-3'	*Nde*I
Pden_*glcDEF*_down	glcDEF_down_fw	5'-AGCCGGGGCGAGC**CATATG**GCTCTCCTCCCTTGGTCGAACGG-3'	*Nde*I
Pden_*glcDEF*_down	glcDEF_down_rv	5'-CTTAAGGCTAGCATGCATCCTAGGCCTGGCGCAGCAACTCGCGGAT-3'	---
mCherry	mCherry_fw	5'-GACACGC**CATATG**GTGAGCAAG-3'	*Nde*I
mCherry	mCherry_rv	5'-GCTACTC**GAATTC**TTACTTGTACAGCTCGTCCATGC-3'	*Eco*RI
Pden_3922/3921_ig	Pden3922_ig_rv	5'-GATAT**GAATTC**TCAGCCGCCGGCACGTCCC-3'	*Eco*RI
Pden_3922/3921_ig	Pden3921_ig_rv	5'-CG**TCTAGA**TCACTCGGGGATGTTGGTCGG-3'	*Xba*I
Pden_4400/4399_ig	Pden4400_ig_rv	5'-GATAT**GAATTC**TCACTGGGCCACGGCCTCG-3'	*Eco*RI
Pden_4400/4399_ig	Pden4399_ig_rv	5'-CTT**TCTAGA**TCACGCACGCGCCAGGATGCC-3'	*Xba*I
Pden_*Pbhc*	Pbhc_fw	5'-CCCTTTTGCGGATTTGAACCG-3'	---
Pden_*Pbhc*	Pbhc_rev-dye[b]	5'-GGAATGAAGATCGGGTTCTGGC-3'	---
Pden_*bhcA*	bhcA_fw	5'-GGGGTCAAATCGGACATCGC-3'	---
Pden_*bhcA*	bhcA_rev-dye[b]	5'-GCGGATGTCGAAGAAGGTGC-3'	---
Pden_*Pglc*	Pglc_fw	5'-CAAGCTCTCCTCCCTTGGTCGAACG-3'	---
Pden_*Pglc*	Pglc_rev-dye[b]	5'-CATGGGCGCTCATGCGGTTGTC-3'	---
Pden_*glcD*	glcD_fw	5'-GCGATAGGCGGTCAGCGCGTCGCATTCATAG-3'	---
Pden_*glcD*	glcD_rev-dye[b]	5'-TTGTCCGGTATTGCCATGCC-3'	---
Pden_*Pglc*[c]	[6FAM]-Pglc_fw	5'-[6FAM]-GGGCGCTCATGCGGTTGTC-3'	---
Pden_*Pglc*[c]	Pglc_rev	5'-GCTCTCCTCCCTTGGTCGAAC-3'	---
-[c]	[6FAM]-tetO_fw	5'-[6FAM]-TCCCTATCAGTGATAGAGA-3'	---
-[c]	tetO_rev	5'-TCTCTATCACTGATAGGGA-3'	---
Pden_*bhcR*	bhcR_104_fw	5'-GTCAGCG**TCTAGA**AATAATTTTGTTTAACTTTAAGAAGG-3'	*Xba*I
Pden_*bhcR*	bhcR_104_rv	5'-GATCACT**GGTACC**TCAGGCTCTTTCGCCGGCATC-3'	*Kpn*I

[a] Nucleotides in bold and underlined are recognition sites for endonuclease restriction enzymes. [b] 5'-labeled with Dyomics 781 fluorescent dye. [c] used to generate fluorescence polarization templates.

References

1. Imam S, Noguera DR, Donohue TJ. CceR and AkgR regulate central carbon and energy metabolism in Alphaproteobacteria. mBio. 2015;6(1).
2. Hermsen R, Okano H, You C, Werner N, Hwa T. A growth-rate composition formula for the growth of *E. coli* on co-utilized carbon substrates. Mol Syst Biol. 2015;11(4):801.
3. Thoma S, Schobert M. An improved *Escherichia coli* donor strain for diparental mating. FEMS Microbiol Lett. 2009;294(2):127-32.
4. Beijerinck MW, Minkman DCJ. Bildung und Verbrauch von Stickoxydul durch Bakterien. Zentralbl Bakteriol Naturwiss. 1910;25:30-63.
5. Schada von Borzyskowski L, Severi F, Kruger K, Hermann L, Gilardet A, Sippel F, et al. Marine Proteobacteria metabolize glycolate via the beta-hydroxyaspartate cycle. Nature. 2019;575(7783):500-4.
6. Stukenberg D, Hensel T, Hoff J, Daniel B, Inckemann R, Tedeschi JN, et al. The Marburg Collection: A Golden Gate DNA Assembly Framework for Synthetic Biology Applications in *Vibrio natriegens*. ACS Synthetic Biology. 2021;10(8):1904-19.
7. Ledermann R, Strebel S, Kampik C, Fischer HM. Versatile Vectors for Efficient Mutagenesis of *Bradyrhizobium diazoefficiens* and Other Alphaproteobacteria. Appl Environ Microbiol. 2016;82(9):2791-9.
8. Schada von Borzyskowski L, Remus-Emsermann M, Weishaupt R, Vorholt JA, Erb TJ. A set of versatile brick vectors and promoters for the assembly, expression, and integration of synthetic operons in *Methylobacterium extorquens* AM1 and other Alphaproteobacteria. ACS Synth Biol. 2015;4(4):430-43.
